# Definitive surgery of primary lesion should be prioritized over preoperative chemotherapy to treat high-grade osteosarcoma in patients aged 41–65 years

**DOI:** 10.1186/s10195-020-00552-w

**Published:** 2020-08-31

**Authors:** Keiko Hayakawa, Seiichi Matsumoto, Keisuke Ae, Taisuke Tanizawa, Yuki Funauchi, Yusuke Minami, Masanori Saito, Atsushi Okawa

**Affiliations:** 1 Department of Orthopedic Oncology, Cancer Institute Hospital of the Japanese Foundation for Cancer Research, 3-8-31, Ariake, Koto-ku, Tokyo, 135-8550 Japan; 2 Department of Orthopedic Surgery, Tokyo Medical and Dental University, Tokyo, Japan

**Keywords:** Osteosarcoma, Definitive surgery, Patient aged 41–65 years

## Abstract

**Background:**

Recently, the number of osteosarcomas in middle-aged and older patients has demonstrated an increasing trend; moreover, their results are comparatively worse than those of young patients. In Europe and the USA, the prognosis for osteosarcoma in middle-aged and older patients has improved with adjuvant chemotherapy. In Japan, however, the prognosis has remained poor.

**Materials and Methods:**

We retrospectively analyzed the outcomes of osteosarcoma, especially in regards to preoperative chemotherapy, from January 1980 to July 2014. A total of 29 patients with high-grade osteosarcoma between the age of 40 and 65 years were included. We included patients without distant metastasis and with primary lesions that were deemed resectable. The mean age was 52.8 years (range 41–65 years), and the mean follow-up period was 103.2 months (range 5–314 months).

**Results:**

Adjuvant chemotherapy was administered to 27 of 29 patients (93%), and 8 of 15 cases (53%) were able to undergo preoperative chemotherapy as planned, including CDDP. A major complication of chemotherapy was acute kidney injury due to CDDP (26%). The 5-year OS and 5-year EFS were 64.9% and 57.1%, respectively. After 2006, a policy to prioritize the resection of the primary lesion was implemented, and if the primary lesion was deemed resectable, preoperative chemotherapy was either not administered or administered for only a short duration. The 5-year OS after 2006 improved to 78.8%.

**Conclusions:**

This study shows that administration of high-dose intensity preoperative chemotherapy was difficult in middle-aged and older patients due to their high rate of acute kidney injury by CDDP. For cases of osteosarcoma in middle-aged and older patients, if the primary lesion is resectable, preoperative chemotherapy should be minimized to prioritize the resection of the primary lesion. It was considered that, with appropriate measures to prevent complications, adjuvant chemotherapy may lead to improved prognosis.

**Level of evidence:**

V.

## Introduction

Although most cases of osteosarcoma occur in young patients, in recent years, both the percentage and total number of middle-aged and older patients over the age of 40 years have demonstrated an upward trend. That is to say, according to the Bone Tumor Registry of Japan, 45 of 162 middle-aged osteosarcoma cases (27.8%) were registered in 2006, while 69 of 207 cases (33.3%) were registered by 2013, accounting for approximately 30% of all cases [1]. As the population continues to age, we expect the number of middle-aged and older patients with osteosarcoma to increase.

For osteosarcoma in young patients, the survival rate has improved with preoperative and postoperative chemotherapy using cisplatin (CDDP), adriamycin (ADM), and methotrexate (MTX) [2–7]. However, middle-aged and older patients are excluded from clinical trials of chemotherapy for osteosarcoma because patients in these age groups may not be able to endure intensive chemotherapy compared with young patients. Since adequate chemotherapy cannot be administered for osteosarcoma in middle-aged and older patients, many studies have reported that their prognosis is poorer compared with that of younger patients [8–20].

Although prognostic factors for osteosarcoma in middle-aged and older patients include its occurrence in the trunk, presence or absence of distant metastasis at first visit, and whether or not the primary site is resectable, these factors are similar to those of younger patients. Adjuvant chemotherapy for osteosarcoma in middle-aged and older patients has been reported to be effective in Europe and the USA. In particular, Bacci et al. reported on adjuvant chemotherapy with ADM, CDDP, and ifosfamide (IFM) on osteosarcoma of the extremities in patients between 41 and 60 years of age and found that the histological response and event-free survival (EFS) were comparable to those of younger patients [9]. In addition, Ferrari et al. reported that similar results were achieved in younger patients in the multicenter EUROpean Bone Over 40 Sarcoma Study (EURO-B.O.S.S.) [20]. However, no reports have demonstrated the efficacy of adjuvant chemotherapy in Japan [14–16]. Few reports have been written on the efficacy of adjuvant chemotherapy in Japan because numerous complications have been associated with chemotherapy, preventing patients from completing their scheduled dose, in addition to the inability to use high-dose MTX. However, previous Japanese reports on osteosarcoma are retrospective multicenter studies, and details on their chemotherapy regimens and complications are unknown, providing limited counsel in terms of improving the outcome of osteosarcoma in middle-aged and older patients. Therefore, we retrospectively analyzed osteosarcoma in middle-aged and older patients treated at a single institution as a measure to improve their treatment outcomes.

## Patients and methods

From January 1980 to July 2014, at our institution, 55 patients between the age of 40 and 65 years at first visit were diagnosed histologically as high-grade osteosarcoma. Among them, 29 cases were included, after excluding the following: those who underwent surgery at a previous hospital and presented with local recurrence after surgery (ten cases), those who could not be treated due to their poor physical condition (one case), those with local progression of primary lesions in the pelvis and skull, which are unresectable (two cases), those who were not initially diagnosed with malignant tumor but were rediagnosed with high-grade osteosarcoma at the time of recurrence (four cases), and those who had distant metastasis at initial diagnosis (nine cases).

The demographic data of our 29 patients are presented in Table 1. The mean age was 52.8 years old (range, 41–65 years). There were 16 males and 13 females. The mean follow-up period was 103.2 months (range, 5–314 months). There were 25 cases of primary osteosarcoma and 4 cases of secondary osteosarcoma. The cause of secondary osteosarcoma was fibrous bone dysplasia in one case, Paget’s disease in one case, and radiation-induced sarcoma in two cases. In terms of primary site, there were seven cases (25%) in the pelvis, five cases (18%) in the proximal and distal femur, and four cases (14%) in the proximal tibia. In addition, there were two cases (6%) in the clavicle and one case (3%) each in the femoral diaphysis, humerus, sternum, radius, fibula, and calcaneus. Pathological subtypes of osteosarcoma were as follows: 22 osteoblastic cases, 5 fibroblastic cases, 1 chondroblastic case, and 1 small cell-type case. According to the UICC TMN staging system, 8 cases were stage IIA and 21 cases were stage IIB.

**Table 1:** Demographic data

Sex	
Male	16
Female	13
Age	52.8 years (41–65 years)
Follow-up period	103.2 months (5–321 months)
Primary/secondary	
Primary	25
Secondary	4
Cause of disease	
Fibrous dysplasia	1
Paget’s disease	1
Radiation induced	2
Primary site	
Pelvis	7
Proximal femur	5
Distal femur	5
Proximal tibia	4
Other	8
Pathological subtype	
Osteoblastic	22
Fibroblastic	5
Chondroblastic	1
Small cell type	1
UICC TMN staging system	
IIA	8
IIB	21

The treatment protocol for osteosarcoma in middle-aged and older patients at our hospital consisted in definitive surgery of the primary lesion if a wide resection for primary tumor could be secured. If a marginal resection was predicted or the histological evaluation of the resected specimen resulted in a marginal resection, combined radiation therapy was administered. Adjuvant chemotherapy was administered when the physical condition of the patient was considered sufficient to undergo chemotherapy. From 1981 to 1983, ADM and cyclophosphamide (CPA) were preoperatively administered intraarterially and intravenously, respectively, while ADM, CPA, and high-dose MTX were postoperatively administered. From 1984 to 1991, CDDP, ADM, high-dose MTX, and a bleomycin, CPA, dactinomycin (BCD) regimen [21] were used. In 1984, CDDP was combined with intraarterial injection therapy but administered only intravenously thereafter. Since 1991, IFM was used in addition to these drugs. From 1984 to 2005, these drugs were administered in three courses before surgery and three courses after surgery. As a result, we found that, when the protocol for young patients with high dose intensity was used for preoperative administration in middle-aged and older patients, there were strong side effects and treatment could not be performed as scheduled. Moreover, more cases occurred in the pelvis in middle-aged and older patients than in younger patients; and because the surgery is more invasive in these cases, a good general condition before surgery is necessary. Based on the above results, the treatment protocol has been modified since 2006 as follows: if preoperative chemotherapy cannot be performed as scheduled due to side effects or if primary surgery is difficult due to ineffective preoperative chemotherapy, priority was given to resection of the primary lesion. If the duration of preoperative chemotherapy was reduced without reducing the total dose of preoperative and postoperative chemotherapy, postoperative chemotherapy was extended. In terms of drugs, we administered either a combination of CDDP and ADM for three courses or each single agent for three courses. In addition, if there was severe pain caused by the primary lesion or activities of daily living (ADL) disorder, surgery was performed first, followed by postoperative chemotherapy.

Considering the possibility of irreversible damage to the kidney due to CDDP, if acute kidney injury was caused by CDDP administration in the first course, we used other drugs without CDDP administration after the second course. Carboplatin (CBDCA) or IFM was used for cases with acute kidney injury due to CDDP or cases with preexisting renal impairment. In those cases, CBDCA and IFM were either used on its own or in combination with etoposide (VP-16). The dosage of each drug used for adjuvant chemotherapy was 100 mg/m^2^/day × 1 day for CDDP, 30 mg/m^2^/day × 2 day for ADM, 2 g/m^2^/day × 5 day for IFM, 8 g/m^2^/day × 1 day for MTX, 100 mg/m^2^/day × 5 day for VP-16, 400 mg/m^2^/day × 1 day for CBDCA; 900 mg/m^2^ for CPA, 1.4 mg/m^2^ (max 2 mg) for VCR, and for BCD regimen, 1.2 mg/m^2^ of bleomycin, 1200 mg/m^2^ of CPA, 40 mg/m^2^ of dactinomycin.

Items for examination included treatment modality, change in adjuvant chemotherapy regimens, reason for the inability to continue preoperative chemotherapy as planned, period to initiation of postoperative chemotherapy, 5-year overall survival (OS), 5-year EFS, and outcome of disease. The surgical margin used for evaluation was based on margins as reported by Kawaguchi et al. Adequate margin was defined as a surgical line at least 1 cm from the reactive zone, and inadequate margin was defined as a surgical line less than 1 cm from the reactive zone [22, 23]. Complications from chemotherapy were assessed based on the common terminology criteria for adverse events (CTCAE) [24]. Creatinine clearance was calculated based on the Cockcroft–Gault equation [Creatinine clearance value = (140 − age)(weight kg)/(72 × Scr) in mL/min, multiplied by 0.85 for females] [25].

In terms of the overall and disease-free survival periods, the Kaplan–Meier method was used to examine the prognostic factors using the log-rank test. *P* value < 0.05 was set as the level of significance. Disease-free and overall survival were defined as either the period up to patient death or last hospital visit. Statistical analyses were performed using JMP software, version 10.1.

## Results

### Treatment

Twenty-one of 29 cases (72%) underwent limb salvage, and 8 cases (28%) underwent amputation. Radiation therapy was preoperatively performed in ten cases (34%). The eight patients who underwent amputation included three cases of hemipelvectomy (two in the pelvis and one in the proximal femur), one case of hip disarticulation in the proximal femur, three cases of above-knee amputations (two in the proximal tibia and one in the distal femur), and one case of below-knee amputation in the calcaneus. The reasons for amputation included the period (1981) in which amputation was the first-choice treatment regardless of the extent of disease in two cases, tumor growth in three cases (one in the proximal tibia and two in the pelvis), pathologic fracture of proximal femur in one case, and infection at the site of tumor in one case of proximal tibia.

Twenty-seven of 29 cases received adjuvant chemotherapy (93%). One patient received only preoperative adjuvant chemotherapy, 3 received only postoperative chemotherapy, and 23 received both preoperative and postoperative chemotherapy. The reason why two patients did not receive chemotherapy was patient refusal.

The results on pre- and postoperative adjuvant chemotherapy are presented in Table 2. Between 1981 and 1983, 4 patients received a chemotherapy regimen that included ADM, CPA, VCR, and MTX; between 1984 and 1991, 4 patients were administered CDDP, ADM, BCD, VCR, MTX, and IFM; and between 1992 and 2005, 11 patients were administered CDDP, ADM, IFM, BCD, and MTX. After 2006, eight patients were administered CDDP and ADM. Eight out of 15 cases (53%) treated between 1984 and 2005 were administered with three courses of preoperative chemotherapy as planned. In seven cases, reasons for failure to administer preoperative chemotherapy on schedule included low performance status (PS 4) because of a pathological fracture in one case, difficulty to continue chemotherapy due to complications in four cases, and difficulty to continue chemotherapy due to tumor growth in two cases.

**Table 2:** Adjuvant chemotherapy pre- and postoperatively

Date (year)	Number of cases	Anticancer drug	Number of cases
1981–1983	4	ADM, CPA, VCR, MTX ADM ADM, CPA	2 1 1
1984–1991	4	ADM, CDDP ADM, CDDP, BCD ADM, CDDP, BCD, VCR.IFM. CDDP, MTX	1 1 1 1
1992–2005	11	ADM, CDDP, IFM, BCD, MTX ADM, CDDP, IFM, BCD ADM, CDDP, IFM, MTX CDDP, BCD, CBDCA CDDP, IFM, MTX CDDP, IFM, BCD ADM, IFM, BCD	2 3 2 1 1 1 1
2006	8	ADM, CDDP ADM, CBDCA ADM, CDDP, CBDCA ADM, CDDP, IFM ADM, CDDP, CBDCA, VP-16 CDDP, CBDCA, VP-16 CBDCA	1 1 1 1 2 1 1
Total	27		27

The ten cases after 2006 (six in the pelvis and one case each in the tibia, calcaneus, radius, and humerus) are presented in Table 3. Seven of ten patients underwent preoperative chemotherapy. Preoperative chemotherapy was administered with the aim of reducing the tumor size in four cases occurring in parts other than the pelvis. Three of four patients underwent surgery (two patients limb salvage and one patient amputation) after one course of chemotherapy because useful findings were not obtained from imaging. The patient whose tumor occurred in the radius and who underwent four courses presented cerebral infarction during a course of CDDP, and the treatment was thus subsequently replaced with CBDCA. Chemotherapy was continued due to its good response, and the limbs were able to be salvaged. Three of the six cases of the pelvis did not undergo preoperative chemotherapy due to an infection at the site of tumor or a large lesion of chondroblastic type, where chemotherapy was considered to be ineffective. Of these three cases, a hemipelvectomy was performed in one case that presented with a particularly advanced local progression; however, the patient died of the disease 22 months after the operation. In two cases, limb-salvage surgery was performed; one patient underwent postoperative adjuvant chemotherapy, and the patients are continuously disease free (CDF) at 74 and 121 months postoperatively. However, of the three remaining cases undergoing preoperative chemotherapy, one patient was referred to our institution after four courses of CDDP and ADM at another hospital because of the unresectable primary tumor. However, we reviewed the case and considered that wide resection of the primary site was achievable. We immediately performed hemipelvectomy, and the patient was alive with disease (AWD) at 30 months postoperatively. In the second case, because imaging showed good response after one course of CDDP and ADM, we proceeded with two additional courses and performed limb-salvage surgery; however, the patient was dead of disease (DOD) at 54 months postoperatively. Since the third case had acute kidney injury after administration of CDDP and ADM, we immediately performed limb-salvage surgery, and there is no evidence of disease (NED) at 65 months postoperatively. In summary, after 2006, seven out of ten patients underwent surgery either because they did not undergo preoperative chemotherapy or because they underwent a single course that was deemed ineffective, and the remaining three patients underwent long-term chemotherapy that included chemotherapy at other hospitals.

**Table 3:** Cases after 2006

	Age (years)	Sex	Primary site	Preoperative adjuvant chemotherapy	Operation	Postoperative adjuvant chemotherapy	Recurrence	Metastasis	Follow-up period	Outcome
1	46	M	Calcaneus	CDDP + ADM(l)	Amputation	CE*(l), CBDCA(3), ADM(l)		+	72	NED
2	65	M	Humerus	CBDCA(I)	Limb salvage	None			80	DOO
3	58	F	Tibia	CDDP + ADM(l)	Limb salvage	CDDP(l), ADM(l), IFM(l)			54	CDF
4	53	M	Radius	CDDP(l), CBDCA(2), CE*(l)	Limb salvage	CE*(3)			66	CDF
*5*	64	F	Pelvis	None	Limb salvage	None			74	CDF
6	44	M	Pelvis	None	Limb salvage	CBDCA(3), ADM(3)			121	CDF
7	59	M	Pelvis	None	Hemipelvectomy	None		+	22	DOD
8	52	M	Pelvis	CDDP + ADM(4)	Hemipelvectomy	CDDP(l)		+	30	AWD
9	45	M	Pelvis	CDDP + ADM(3)	Limb salvage	CDDP + ADM(I), CDDP(I), CE*(2)		+	54	DOD
10	63	M	Pelvis	CDDP + ADM(l)	Limb salvage	CBDCA(3), ADM(2)		+	68	NED

### Local recurrence

Local recurrence was found in 1 of 29 cases (3%). The primary site in case of local recurrence was proximal fibula. Stump recurrence was observed at 12 months postoperatively in this case, despite surgical margin having been histologically assessed as an adequate margin.

### Overall survival

**Fig. 1:**
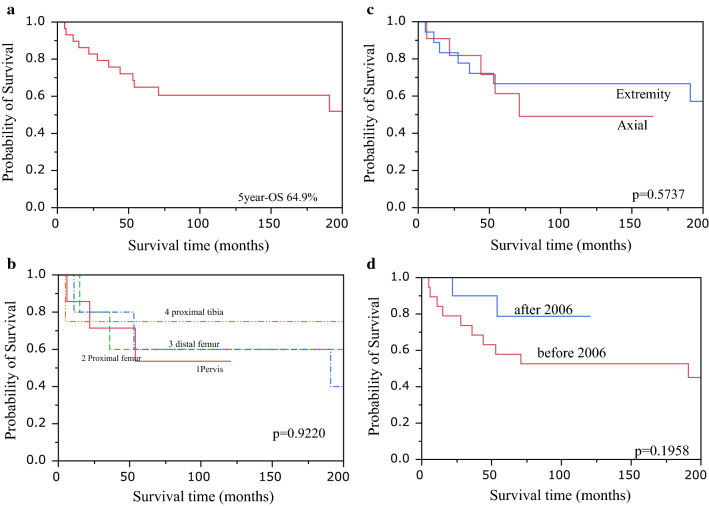
Overall survival. **a** The 5-year overall survival (OS) was 64.9%. **b** The 5-year survival rates for each location were 53.6% for the pelvis, 60.0% for the proximal femur, 60.0% for the distal femur, and 75.0% for the proximal tibia. **c** There was no significant difference between the 5-year survival rate for the trunk at 61.4% and that for the extremities at 66.7% (log-rank test, *P* = 0.5737). **d** The 5-year survival rate improved to 78.8% from 57.9% (log-rank test, *P* = 0.1958)

The 5-year OS was 64.9% (Fig. 1a). The 5-year survival rates for each location were 53.6% for the pelvis, 60.0% for the proximal femur, 60.0% for the distal femur, and 75.0% for the proximal tibia (Fig. 1b). There was no significant difference between the 5-year survival rate for the trunk at 61.4% and that for the extremities at 66.7% (log-rank test, *P* = 0.5737) (Fig. 1c).

When comparing the ten cases after 2006 in which resection of the primary tumor was prioritized to determine the administration period of preoperative chemotherapy (6 cases in the pelvis and 1 case each in the tibia, calcaneus, radius, and humerus) with the preceding 19 cases (11 cases in the femur, 3 cases in the tibia, 2 cases in the clavicle, and 1 case each in the pelvis, sternum, and fibula), the 5-year survival rate improved to 78.8% from 57.9% (log-rank test, *P* = 0.1958) (Fig. 1d). The amputation rate was 30% after 2006 and 26% prior to 2006.

### Event-free survival

**Fig. 2:**
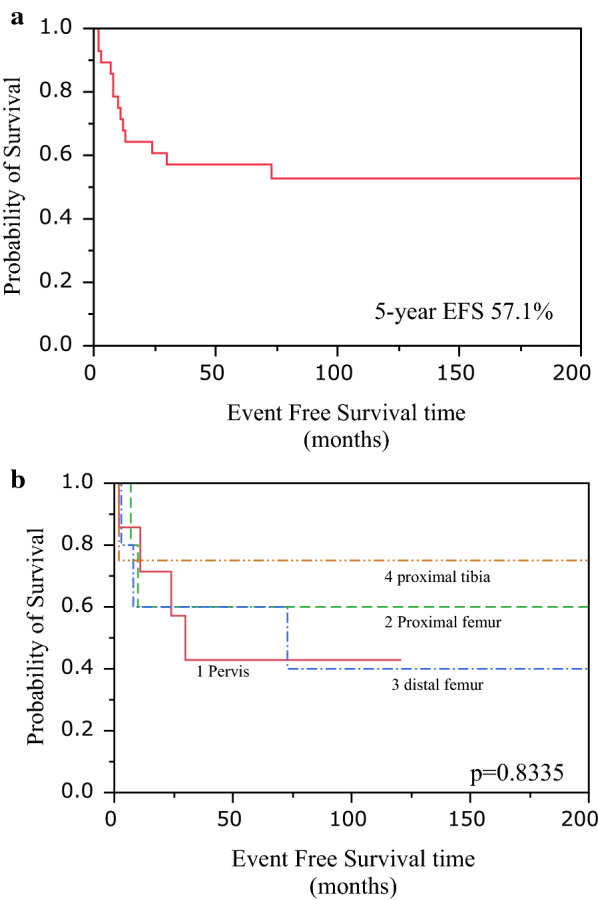
Event-free survival. **a** The 5-year overall EFS was 57.1%. **b** The EFS for each location was 42.9% for the pelvis, 60.0% for the proximal femur, 60.0% for the distal femur, and 75.0% for the proximal tibia. Although the EFS of the pelvis was slightly low, no significant difference was observed (log-rank test, *P* = 0.8335)

The 5-year overall EFS was 57.1% (Fig. 2a). The EFS for each location was 42.9% for the pelvis, 60.0% for the proximal femur, 60.0% for the distal femur, and 75.0% for the proximal tibia. No significant difference was observed (Fig. 2b) (*P* = 8335).

### Complications from chemotherapy

In 19 of 27 patients, CDDP was administered in combination with a single drug or ADM, of which 5 patients (26.3%) showed nephrotoxicity due to CDDP. Nephrotoxicity was grade 1 in two cases and grade 2 in three cases; and creatinine elevation was grade 1 in three cases and grade 2 in two cases. Of these five cases, four were in their 40s and one was in their 50s. Four of five cases exhibited elevated creatinine levels from the first course. Hepatic toxicity (grade 4) was observed in 1 of 20 patients (5%) who received a single drug or ADM. IFM was administered in nine cases, and disturbance of consciousness (grade 3) was observed in one case with no injury to the kidney. There were no mortalities due to chemotherapy.

### Outcome of disease

The outcome of disease was CDF in ten cases, AWD in one case, NED in two cases, DOD in 12 cases, and death of other factors (DOO) in four cases.

## Discussion

As the population ages in Japan, both the percentage and total number of osteosarcomas in middle-aged and older patients increase on an annual basis [1]. Previous domestic reports on osteosarcoma of middle-aged and older patients have mostly been multicenter studies [14–16], and single-institution reports remain scarce in literature [18].

In younger patients, the sites of predilection for osteosarcoma are the distal femur, proximal tibia, and humerus. In contrast, a large percentage of occurrence in the trunk of middle-aged and older patients is reported in literature, ranging from 19% to 49% [8–19]. This higher incidence is due to the causes of secondary sarcoma’s, such as Paget’s disease and radiation-induced sarcoma, being common in the pelvis [10–12].

In this study, ten cases (33%) were located in the trunk, demonstrating a comparable tendency to that of previous reports. More cases were located in the proximal femur in this study compared with other institutions, with 5 of 29 cases (18%) each in the proximal and distal femur and 4 cases in the proximal tibia (14%). Though the number of reported incidence in the distal femur was higher than that in the proximal femur in Japan and other countries, our results were different [8–18].

In terms of the efficacy of adjuvant chemotherapy, many studies from the USA and Europe report adjuvant chemotherapy to be effective [7–18]. In 2007, Bacci et al. administered adjuvant chemotherapy to patients between the age of 41 and 60 years with a dosage intensity equivalent to what is administered in younger patients, and in 34 cases without metastasis occurring in the extremities, they reported a 70% 5-year survival rate and suggested that adjuvant chemotherapy was effective [9]. In 2003, Grimer et al. reported the results of a retrospective multicenter study at the European Musculo Skeletal Oncology Society (EMSOS). A total of 238 cases between the age of 40 and 60 years consisting of high-grade cases occurring in the extremities without metastasis were included in the study. Among them, 69% were administered adjuvant chemotherapy, and they reported that adjuvant therapy was effective, with a 5-year OS of 51% in the chemotherapy group, 39% in the nonchemotherapy group, and 46% in the average between the two groups [12]. In a 2018 EUROpean Bone Over 40 Sarcoma Study (EURO-B.O.S.S.), Ferrari et al. reported that, in 218 patients over 40 years of age with or without metastasis undergoing a regimen equivalent to protocols for younger patients, an aggressive approach with chemotherapy and surgery led to results that were comparable to those of younger patients. In the USA and Europe, adjuvant chemotherapy was effective in improving the prognosis, even in multicenter studies where chemotherapy regimens were not consistent.

However, in Japan and Asian countries, no improvements in prognosis have been reported as a result of adjuvant chemotherapy, with poor prognosis in middle-aged and older patients compared with that of the chemotherapy-administered group in American and European reports. Iwata et al. retrospectively investigated 86 high-grade osteosarcomas in patients over 40 years of age and reported that adjuvant chemotherapy did not improve prognosis, with a 5-year survival rate in cases with N0M0 of 48.3% [13]. In addition, Joo et al. described a multicenter study (Eastern Asian Musculoskeletal Oncology Group) and reported that the 5-year survival rate for their 232 cases with N0M0 was 59.4%, and no effect on prognosis was reported with adjuvant chemotherapy [19]. Despite the use of key drugs for osteosarcoma, including CDDP, ADM, MTX, and IFM, being identical between Asian and Western countries [2–10], the reason for the discrepancy in results may be a result of differences in the dose of chemotherapy. Although some of these reports do not provide details on chemotherapy, considering that cases that achieved a similar protocol to that of younger patients exhibited good prognosis, insufficient administration of chemotherapy is a highly probable cause for poor prognosis. In middle-aged and older patients, myelosuppression and nephrotoxicity can affect the dose of chemotherapy, but renal disorder is irreversible and more severe than myelosuppression. In 2005, Manoso et al. reported grade 2 chronic nephrotoxicity in 11 of 58 cases (17%) with osteosarcoma in patients over 40 years of age [11]. In 2007, Bacci et al. reported grade 1–2 chronic nephrotoxicity in only 0.6% of the 34 cases with osteosarcoma between the age of 41 and 60 years [9]. In this study, nephrotoxicity occurred in 5 out of 19 patients (26.3%) who were administered CDDP. No nephrotoxicity related to IFM was observed. According to a report by Manoso et al., the dosage of CDDP or MTX was not described due to disparities in chemotherapy regimens. In a report by Bacci et al., a regimen consisting of a total of 12 courses with three drugs, namely CDDP, ADM, and IFM, was administered to patients, who underwent eight courses of CDDP at 120 mg/m^2^. Although 50% of cases were administered with a dose intensity greater than 80%, the nephrotoxicity associated with CDDP was only 0.6% [9], greatly differing from our results.

However, in the EURO BOSS regimen, the total dose of CDDP is set at a maximum of 600 mg/m^2^. The dose reduction criteria of CDDP involves a 75% reduction when creatinine is at 1.2 mg/dl or more, and when there is repeated renal failure, CDDP administration is discontinued. Nephrotoxicity was observed in 48 cases (28%), but most were grade 1. Grade 3 creatinine elevation was observed in two patients, and grade 4 nephrotoxicity was observed in one patient, who subsequently started dialysis therapy. The incidence of kidney injury in each regimen was 5% in CDDP/ADM, 13% in IFM/ADM, and 8% in IFM/CDDP. When comparing these results with the current study, the incidence of kidney injury appears to be comparable, while kidney injury due to CDDP was more common (26%).

In terms of CDDP, discrepancy in the incidence of renal impairment due to race differences have been reported [26, 27], but there are no reports comparing Asian and Western populations. In this study, the Asian population was more likely to exhibit nephrotoxicity compared with the Western population, which may have led to the inability to administer enough adjuvant chemotherapy to improve prognosis. Although adjuvant chemotherapy for osteosarcoma in younger patients is an indispensable treatment, there is no consensus as to whether this should be administered before or after surgery [20]. Reasons to perform preoperative chemotherapy include (1) drug susceptibility, (2) limb salvage by way of downstaging, and (3) treatment for micrometastatic lesions. In our study, no cases achieved limb salvage by downstaging with preoperative chemotherapy; and in highly malignant osteosarcoma cases in patients between the ages of 40 and 65 years, we believe that a level of limb salvage by downstaging equivalent to that in younger patients cannot be expected.

Since 2006, the treatment protocol has been modified from a conventional method of administering a fixed amount of chemotherapeutic agent before surgery to a policy that prioritizes the resection of primary lesions and places its focus on postoperative chemotherapy. The 5-year survival rate improved to 78.8% after the implementation of the modified treatment protocol. We believe that the accuracy of decision-making regarding the resectability of osteosarcoma may greatly affect prognosis, and an individually tailored timing of surgery is important in determining whether a curative resection is possible for osteosarcoma in middle-aged and older patients.

Pelvis location is considered a poor prognostic factor for mature osteosarcoma [14, 15], and it is often difficult to determine whether primary tumor can be resected prior to treatment. In this study, there were 13 M0 cases from November 1978 to July 2014. Of them, ten patients (77%) were deemed possible to undergo resection for the primary lesion. There were no cases in which preoperative treatment was successful, wherein a primary lesion that was considered difficult to resect became resectable. However, even if primary resection was considered achievable, there was no difference in the 5-year survival rate for tumors occurring in the extremities and those occurring in the trunk; thus, we believe that the ability to determine whether the resection of a primary tumor is achievable is very important.

In this study, elderly patients in Japan were susceptible to acute kidney injury due to CDDP; therefore, it was difficult to perform intensive preoperative chemotherapy and expect a tumor response. The problems involving pelvic occurrences, which are common in middle-aged patients with osteosarcoma, are summarized below. First, postoperative chemotherapy is often delayed due to protracted wound healing after surgery, which can be addressed by providing long-term chemotherapy that incorporates its postoperative dosage in the preoperative dosage. Second, if the primary tumor is large, wide resection of tumor becomes difficult when the preoperative chemotherapy is ineffective. The response to this problem is to focus on postoperative chemotherapy rather than preoperative chemotherapy and to perform surgery on the primary lesion when this is resectable. In middle-aged and older patients, it is also necessary to overcome the difficulty of performing high-dose-intensity preoperative chemotherapy. For osteosarcoma in pelvis in middle-aged patients, we believe that a treatment strategy which can respond to a variety of situations is necessary compared with cases that occur in the extremities of younger patients.

Prospective research is indispensable in the future, as the results of our study were limited by the fact that chemotherapy regimens have varied with time. Adjuvant chemotherapy is indispensable for improving survival in middle-aged and older patients with osteosarcoma. In Japan, however, a treatment system different from that of young patients should be considered because acute kidney injury due to CDDP is more likely to occur.

## Data Availability

The datasets and/or analyses of the current study are available from the corresponding author on reasonable request.

## References

[1] Japan Orthopaedic Association Musculoskeletal Tumor Committee/National Cancer Center. (2013) Bone and Soft Tissue Tumor Registry in Japan

[2] Kudawara I, Aoki Y, Ueda T, Araki N, Naka N (2013). Neoadjuvant and adjuvant chemotherapy with high-dose ifosfamide, doxorubicin, cisplatin and high-dose methotrexate in non-metastatic osteosarcoma of the extremities: a phase II trial in Japan. J Chemother.

[3] Meyers PA, Gorlick R, Heller G, Casper E, Lane J, Huvos AG (1998). Intensification of preoperative chemotherapy for osteo- genic sarcoma: results of the Memorial Sloan-Kettering (T-12) protocol. J Clin Oncol.

[4] Lewis IJ, Weeden S, Machin D, Stark D, Craft AW (2000). Received dose and dose-intensity of chemotherapy and outcome in nonmetastatic extremity osteosarcoma. J Clin Oncol.

[5] Wilkins RM, Cullen JW, Odom L, Jamroz BA, Cullen PM, Fink K (2003). Superior survival in treatment of primary nonmetastatic pediatric osteosarcoma of the extremity. Ann Surg Oncol.

[6] Aljubran AH, Griffin A, Pintilie M, Blackstein M (2009). Osteosarcoma in adolescents and adults: survival analysis with and without lung metastases. Ann Oncol.

[7] Iwamoto Y, Tanaka K, Isu K, Kawai A, Tatezaki S, Ishii T (2009). Multiinstitutional phase II study of neoadjuvant chemotherapy for osteosarcoma (NECO study) in Japan: NECO-93 J and NECO-95. J J Orthop Sci.

[8] Bacci G, Ferrari S, Mercuri M, Longhi A, Fabbri N, Galletti S, Forni C, Balladelli A, Serra M, Picci P (2007). Neoadjuvant chemotherapy for osteosarcoma of the extremities in patients aged 41–60 years: outcome in 34 cases treated with adriamycin, cisplatinum and ifosfamide between 1984 and 1999. Acta Orthop.

[9] Bacci G, Ferrari S, Donati D, Longhi A, Bertoni F, Di Fiore M, Comandone A, Cesari M, Campanacci M (1998). Neoadjuvant chemotherapy for osteosarcoma of the extremity in patients in the fourth and fifth decade of life. Oncol Rep.

[10] Bacci G, Longhi A, Bertoni F, Bacchini P, Ruggeri P, Versari M, Picci P (2005). Primary high-grade osteosarcoma: comparison between preadolescent and older patients. J Pediatr Hematol Oncol.

[11] Manoso MW, Healey JH, Boland PJ, Athanasian EA, Maki RG, Huvos AG, Morris CD (2005). De novo osteogenic sarcoma in patients older than forty: benefit of multimodality therapy. Clin Orthop Relat Res.

[12] Grimer RJ, Cannon SR, Taminiau AM, Bielack S, Kempf-Bielack B, Windhager R, Dominkus M, Saeter G (2003). Osteosarcoma over the age of forty. Eur J Cancer.

[13] Carsi B, Rock MG (2002). Primary osteosarcoma in adults older than 40 years. Clin Orthop Relat Res.

[14] Iwata S, Ishii T, Kawai A, Hiruma T, Yonemoto T, Kamoda H, Asano N, Takeyama M (2014). Prognostic factors in elderly osteosarcoma patients: a multi-institutional retrospective study of 86 cases. Ann Surg Oncol.

[15] Nishida Y, Isu K, Ueda T (2009). Osteosarcoma in the elderly over 60 years: a multicenter study by the Japanese Musculoskeletal Oncology Group. J Surg Oncol.

[16] Okada K, Hasegawa T, Nishida J, Ogose A, Tajino T, Osanai T, Yanagisawa M, Hatori M (2004). Osteosarcomas after the age of 50: a clinicopathologic study of 64 cases–an experience in northern Japan. Ann Surg Oncol.

[17] Song WS, Kong CB, Jeon DG, Cho WH, Kim MS, Lee JA, Yoo JY, Kim JD, Lee SY (2010). Prognosis of extremity osteosarcoma in patients aged 40–60 years: a cohort/case controlled study at a single institute. Eur J Surg Oncol.

[18] Urakawa H, Tsukushi S, Sugiura H, Yamada K, Yamada Y, Kozawa E, Arai E, Futamura N, Ishiguro N, Nishida Y (2014). Neoadjuvant and adjuvant chemotherapy with doxorubicin and ifosfamide for bone sarcomas in adult and older patients. Oncol Lett.

[19] Joo MW, Shin SH, Kang YK, Kawai A, Kim HS, Asavamongkolkul A, Jeon DG, Kim JD (2015). Osteosarcoma in Asian populations over the age of 40 years: a multicenter study. Ann Surg Oncol.

[20] Ferrari S, Bielack SS, Smeland S et al (2018) EURO-B.O.S.S.: A European study on chemotherapy in bone- sarcoma patients aged over 40: outcome in primary high-grade osteosarcoma: Tumori 104(1):30–36 10.5301/tj.5000696 29218692

[21] Mosende C, Gutierrez M, Caparros B (1977). Combination chemotherapy with bleomycin, cyclophosphamide and dactinomycin for the treatment of osteogenic sarcoma. Cancer.

[22] Kawaguchi N, Matsumoto S, Manabe S (1995). New method of evaluating the surgical margin and safety margin for musculoskeletal sarcoma analysed on the basis of 457 surgical cases. J Cancer Res Clin Oncol.

[23] Kawaguchi N, Ahmed AR, Matsumoto S, Manabe J, Matsushita Y (2004). The concept of curative margin in surgery for bone and soft tissue sarcoma. Clin Orthop Relat Res.

[24] National Cancer Institute: National Cancer Institute Common Toxicity Criteria version 4.0. (2013) Accessed September 10

[25] Cockcroft DW, Gault MH (1976). Prediction of creatinine clearance from serum creatinine. Nephron.

[26] Bhat ZY, Cadnapaphornchai P, Ginsburg K (2015). Understanding the risk factors and long-term consequences of cisplatin-associated acute kidney injury: an observational cohort study. PLoS ONE.

[27] Goorin AM, Schwartzentruber DJ, Devidas M (2003). Presurgical chemotherapy compared with immediate surgery and adjuvant chemotherapy for nonmetastatic osteosarcoma: Pediatric Oncology Group Study POG-8651. J Clin Oncol.

